# Relative Risk Prediction of Norovirus Incidence under Climate Change in Korea

**DOI:** 10.3390/life11121332

**Published:** 2021-12-02

**Authors:** Tae-Kyoung Kim, Jayeong Paek, Hwang-Yong Kim, Ilsu Choi

**Affiliations:** 1 Department of Mathematics and Statistics, Chonnam National University, Gwangju 61186, Korea; 178925@chonnam.ac.kr (T.-K.K.); 196790@chonnam.ac.kr (J.P.); 2 Microbial Safety Division, National Institute of Agricultural Sciences, Rural Development Administration, Wanju 55365, Korea; hykim@korea.kr

**Keywords:** norovirus, climate change, relative risk, generalized additive logistic model

## Abstract

As incidences of food poisoning, especially norovirus-induced diarrhea, are associated with climate change, there is a need for an approach that can be used to predict the risks of such illnesses with high accuracy. In this paper, we predict the winter norovirus incidence rate in Korea compared to that of other diarrhea-causing viruses using a model based on B-spline added to logistic regression to estimate the long-term pattern of illness. We also develop a risk index based on the estimated probability of occurrence. Our probabilistic analysis shows that the risk of norovirus-related food poisoning in winter will remain stable or increase in Korea based on various Representative Concentration Pathway (RCP) scenarios. Our approach can be used to obtain an overview of the changes occurring in regional and seasonal norovirus patterns that can help assist in making appropriate policy decisions.

## 1. Introduction

As the reproduction of microorganisms is substantially affected by weather-related factors such as temperature and humidity, climate change is extremely likely to cause changes in the seasonal patterns of food poisoning incidents caused by microorganisms. Therefore, as climate conditions continue to change, it is expected that adjustments to food safety policies related to food will become necessary, and scientific methodologies that can accurately predict long-term fluctuations in food poisoning patterns must be developed to assist administrators in proactively devising reasonable policies to respond to the effects of climate change. Most advanced countries already have access to vast amounts of weather and food poisoning-related clinical data; the key challenge is deriving useful information from these big data. According to a recent 2016 study, more than 40 million disability-adjusted life years were lost in children under the age of five due to cases of diarrhea and related deaths [1]. Although the incidence of diarrhea may be reduced by economic development, some reports have found that climate change damages urban infrastructure and reduces overall water availability [2]. The spread of diarrhea is complex and depends on many factors. Infectious diarrhea can be caused by a variety of pathogens, and it is affected by both host susceptibility and environmental components. Therefore, there is an urgent need for research that quantifies the impact of diarrheal illness as well as improved predictive estimates [3,4,5,6]. This paper chooses to consider norovirus specifically for two main reasons. First, variations in the incidence pattern of norovirus are clinically significant. As diagnostic technology continues advancing, norovirus has been increasingly identified as a major cause of food poisoning [7]. The World Health Organization (WHO) reports that norovirus is one of the top five causes of death from food poisoning [8]. More than 200,000 people die from norovirus every year, including 70,000 children in developing countries [9]. In the United States, norovirus causes 23 million cases of acute gastroenteritis (AGE) every year [10]. According to the Centers for Disease Control and Prevention (CDC), norovirus causes annual economic losses of more than $2 billion in the United States alone. In other words, norovirus continues to have a negative impact on both human life and the economy worldwide. Second, norovirus has a distinct seasonal pattern, with the frequency of cases rising in the winter [11]. Because of this, it is also called “the winter vomiting disease” [12,13]. New norovirus variants often emerge on cruise ships in the winter and early spring. In the United States, there were particularly high numbers of deaths from norovirus in the winter months in both 2002–2003 and 2006–2007. These seasonal patterns in disease prevalence make it easier to analyze the relationship between weather-related factors and food poisoning using probabilistic methods. As described above, the main causes of norovirus are contaminated food and human contact; however, many papers examining the relationship between norovirus incidence and climate support the assertion of this paper. Previous studies have shown that norovirus is negatively correlated with mean temperature [14] and that it is a wintertime phenomenon, at least in the temperate northern hemisphere [15]. Another study reviewing norovirus incidence over the previous decade showed differences in case numbers between influenza and norovirus infections, with norovirus still showing strong incidence in certain high population density areas [16]. A recent study examined temperature and relaxation rates among patients with diarrhea given the climate change scenarios in Japan. That study estimated the future probability of the disease based on a simple comparison by region [17]. The above study explains the importance of temperature-related future changes in diarrheal patients. To simplify the model and actively identify temperature-related effects, among various weather-related factors, the current study focused on average daily temperature among various weather-related factors. In this paper, we also attempted to predict the variations in norovirus incidence that will occur as the temperature continues to rise in winter due to climate change. 

We introduce an index that we developed to quantify the incidence rate of norovirus-induced diarrhea according to Representative Concentration Pathway (RCP) scenarios that explain climate change, and we used this index to calculate the future risk of disease due to climate change. In this analysis, a generalized additive linear model (GALM) method using B-spline was used to compensate for the fact that the probability of dependence on the explanatory variable increases or decreases in simple logistic regression. In addition, the risk assessment was made more objective by using the relative risk index (RRI) to represent risk.

## 2. RCP Scenarios

In order to establish an effective response and adaptation plan for risk factors that will appear due to climate change, the Intergovernmental Panel on Climate Change (IPCC) introduced a set of climate change scenarios. The RCP scenarios consider both recent observations along with greenhouse gas reduction technologies. The IPCC has recommended that researchers use the RCP scenarios as basic data for studies that involve climate change impact evaluations; in particular, four scenarios have been introduced: RCP2.6, RCP4.5, RCP6.0, and RCP8.5. The numbers in the scenario names indicate the increasing amounts of net radiation energy experienced by the year 2100 [18]. 

The Korea Meteorological Administration (KMA) has contributed to the RCP scenarios (http://www.climate.go.kr (accessed on 5 August 2021)). The KMA developed RCP scenarios using five regional climate models (HadGEM3-RA, RegCM4, SNURCM, GRIMs, WRF) [19,20,21,22,23]. As climate models contain uncertainty associated with several sources, e.g., imprecise initial conditions, some statistical refinement techniques and bias correction methods are applied to the output of the model, before providing data to the end users [24]. From these five models they created two model ensembles. The first model is MME4s, based on data from HadGEM3-RA, RegCM4, SNURCM, and WRF. The second one is MME5s, based on data from HadGEM3-RA, RegCM4, SNURCM, GRIMs, and WRF. Climate projections until the year 2100 are evaluated under RCP2.6 and 6.0 for MME4s, and under RCP4.5 and 8.5 for MME5s. The latitude and longitude of the RCP scenario data range from 33° N to 39° N and 124.5° E to 132.0° E, respectively, which includes the entirety of the southern Korean Peninsula.

## 3. Data Descriptions

**Figure 1:**
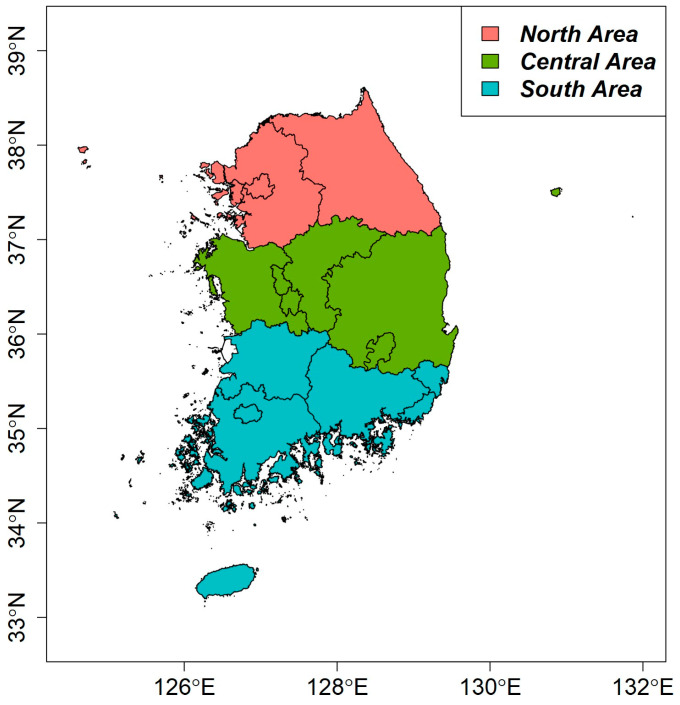
Three study areas in the South Korean Peninsula separated based on latitude and administrative divisions.

From 2005 to 2018, the total number of patients in Korea reported to have diarrhea was 283,651. Of these cases, 24,642 were caused by norovirus, representing a rate of 8.69%, thus making norovirus a very significant cause of diarrhea. These clinical data come from the Korea Centers for Disease Control and Prevention Portal (http://www.kdca.go.kr/ (accessed on 3 September 2020)). Table 1 summarizes the known norovirus outbreaks by gender and age and the rate of norovirus diarrhea cases compared to diarrhea cases from all-causes. The incidence rates of norovirus do not significantly differ by gender, with the rates in males and females being 8.82% and 8.53%, respectively. In contrast, the effect of age is clearly significant. In particular, the incidence rates of norovirus in the age groups of 0–5, 6–15, 16–59, and over 59 are 13%, 3.37%, 4.79%, and 8.74%, respectively. This shows that very young people are significantly more vulnerable to norovirus than those in other age groups. When dividing Korea into northern, central, and southern regions, there were slight differences in the rates of norovirus outbreaks between regions, with rates of 10.14%, 6.05%, and 9.26%, respectively. In the southern part of the country along the coast, diet and temperature are expected to affect the risk of norovirus outbreaks. The northern part of the country also has a somewhat high risk as it includes Seoul, which is particularly densely populated, leading to higher disease risk. The results of the logistic regression analysis used to calculate the odds ratio by gender, age, and region for norovirus occurrence are shown in Table 2. The odds ratio for a female (vs. male) is 0.101, which is not significant. Age had a significant effect: compared to those aged 0–5, the odds ratios were 0.646, 0.350, and 0.245 for individuals aged 6–15, 16–59, and 60 and older, respectively. This indicates that individuals under five years of age are substantially more vulnerable to norovirus infection than those in other age groups. In addition, the odds ratio for the central and southern regions of the country relative to the northern region were significantly different at 0.777 and 1.062, respectively. Figure 1 shows the regions described in Table 2 on a map for better understanding. We used R 4.2.1 for all data analysis [25,26,27,28,29,30].

## 4. Methods

First, the relationship between weather-related factors and the occurrence of food poisoning caused by norovirus was analyzed stochastically. Based on the results of that analysis, we calculated how the seasonal pattern of norovirus incidence is expected to change in the future due to climate change. In Korea, incidences of diarrhea are reported daily by region and cause. We used average daily temperature data from the Meteorological Data Open Portal (https://data.kma.go.kr/ (accessed on 5 August 2021)). The number of diarrhea patients with norovirus may vary as winter temperatures rise rapidly in accordance with the climate projections under the considered RCP scenarios.

In addition, a generalized additional model (GAM) was employed to express the additive model of the functions including these bases. The logistic model using GAM helps us to predict the probability of the occurrence of norovirus according to the average temperature. In the GAMs that were used, the linear components $\beta_{j}\sum x_{ij}\;$of the model were replaced with $\beta_{j}\sum f_{j}\left(x_{ij}\right)$ [31,32,33,34]. $\pi (y_{i}|x_{i1},x_{i2},\cdots ,x_{ip})$ is used to denote the probability that norovirus cases appear along with the explanatory variables $x_{i1},x_{i2},\cdots ,x_{ip}$. The GALM assumes that

(1) $$log\left[\frac{\pi \left(y_{i}|x_{i1},x_{i2},\ldots ,x_{ip}\right)}{1-\pi \left(y_{i}|x_{i1},x_{i2},\ldots ,x_{ip}\right)}\right]=\beta_{0}+\beta_{1}f_{1}\left(x_{i1}\right)+\beta_{1}f_{1}\left(x_{i1}\right)+\cdots +\beta_{p}f_{p}\left(x_{ip}\right)\;$$

where we used the functions $f_{1},f_{2},\cdots ,f_{p}$ to smooth bases estimated in various forms, such as with B-spline, cubic splines, and natural cubic splines. We used recursive 4th B-spline bases as supports of the mean temperature as follows [34,35,36,37,38]. Let U be a set of m+1 non-decreasing numbers, $u_{0}\leq u_{1}\leq u_{2}\leq \cdots \leq u_{m}$.

$$N_{i,0}\left(u\right)=\{\begin{array}{c} 1\;if\;u_{i}\leq u\leq u_{i+1}\\ 0\;\mathrm{otherwise}\; \end{array}$$

(2) $$N_{i,p}\left(u\right)=\frac{u-u_{i}}{u_{i+p}-u_{i}}N_{i,p-1}\left(u\right)+\frac{u_{i+p+1}-u}{u_{i+p+1}-u_{i+1}}N_{i+1,p-1}\left(u\right)$$

We create a logistic regression model using the previously generated $N_{i,p}$ as $f_{p}\left(x_{ip}\right)$ and the presence or absence of norovirus as the dependent variable. A backfitting algorithm is typically used for estimations when the splines are complex. However, in this paper, the splines are calculated and then used to estimate the logistic regression because they can be calculated with relative ease. We calculate the binary logistic regression model using iteratively reweighted least squares (IRLS), which is equivalent to maximizing the log-likelihood of a Bernoulli distributed process using Newton’s method as follows.

$$\mathrm{parameters}:\;w^{T}=\left[\beta_{0},\beta_{1},\beta_{2},\ldots \right]$$

$$\mathrm{explanatory}\;\mathrm{variables}:\;x\left(i\right)={\left[\beta_{0},\beta_{1},\beta_{2},\ldots \right]}^{T}$$

$$X=\left[\begin{array}{ccc} 1 & x_{1}\left(1\right) & x_{2}\left(1\right)\\ 1 & x_{1}\left(2\right) & x_{2}\left(2\right)\\ \vdots & \vdots & \vdots \end{array}\;\begin{array}{c} \cdots \\ \cdots \end{array}\right]$$

$$\mathrm{response}\;\mathrm{variables}:\;y\left(i\right)={\left[y\left(1\right),y\left(2\right),\ldots \right]}^{T}$$

$$\mathrm{expected}\;\mathrm{value}\;\mathrm{of}\;\mathrm{the}\;\mathrm{Bernoulli}\;\mathrm{distribution}:\;\mu \left(i\right)=\frac{1}{1+e^{-w^{T}x\left(i\right)}}$$

μ=[μ(1),μ(2),…]

using iterative algorithm

(3) $$w_{k+1}={\left(X^{T}S_{K}X\right)}^{-1}X^{T}\left(S_{k}Xw_{k}+y-\mu_{k}\right)$$

The average temperature that maximizes the probability of GALM is defined as the maximum rate temperature, and the intervals of height corresponding to 0.8 and 0.9 times the maximum rate are respectively defined as the risk interval and the high-risk interval.

We employed the RRI to understand the effect of temperature-dependent probabilities in the RCP scenarios. RRI measures how the probability of occurrence is driven by temperature in GALM models.

(4) $$\mathrm{Relative}\;\mathrm{risk}\;\mathrm{index}\;\left(\mathrm{RRI}\right)=\sum_{y_{i}\in A}Deviation_{i}\times \chi \left(Deviation_{i}\right)$$

where A is defined as the focus set, $Deviation_{i}$ is defined as $\pi (y_{i}|\cdot )$—the critical value, and χ represents the indicator function.

Larger RRIs indicate an increased risk of infection by norovirus. That is, the probability in a particular group is greater than the mean probability of being infected by norovirus among diarrhea patients. For example, the incidence of norovirus among all diarrhea patients aged 0–5 is 13.00%, so the critical value is 0.13. We can compute the RRI for age, region, and X-year RCP scenarios.

**Figure 2:**
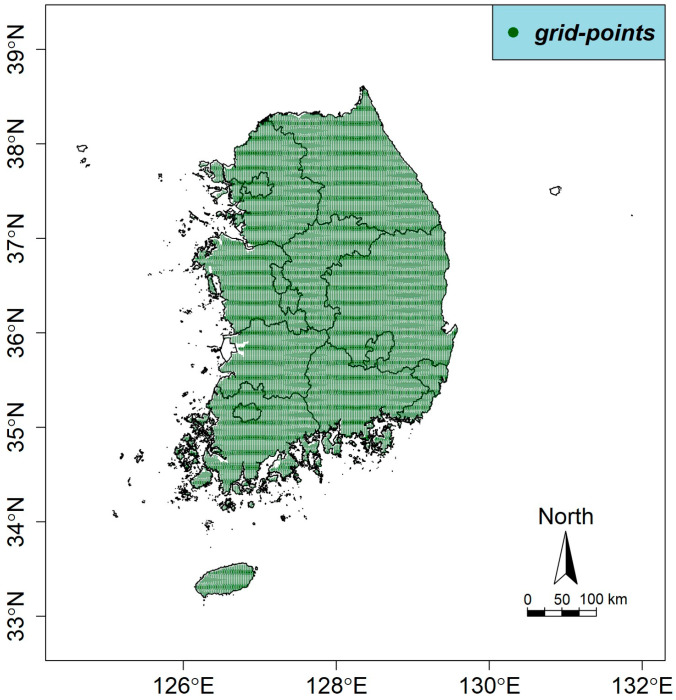
The 96,172 grid points based on the land of the southern Korean peninsula to calculate the RRIs and the GALM model.

We calculated the RRI in each RCP scenario for the winter months in Korea (December to February). We organized the 451,351 grids in the RCP scenarios into 96,172 grids based on their location in the Korean peninsula to calculate the RRIs, and then we used the GALM model to predict the probability of the incidence of norovirus on a daily basis. Figure 2 shows the grid, made up of 96,172 points, we used. In a logistic regression model the outcome of $y_{i}$ is 0 or 1, with 1 indicating an event occurring (such as becoming infected with a disease) and 0 indicating no event occurring. In a typical logistic model, the probability of a response increases or decreases depending on the value of the explanatory variable. Therefore, the quadratic form, which increases and decreases in order to obtain an optimum value, is not applicable here. To compensate for this, a number of orthogonal bases were used in this paper to make the quadratic form feasible.

## 5. Results

**Figure 3:**
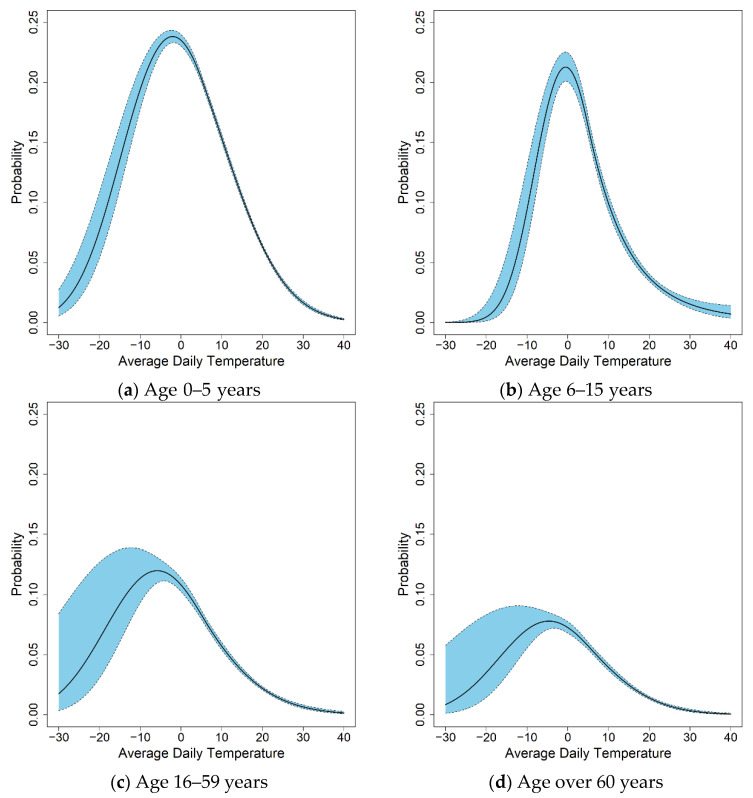
Predicted norovirus incidence rates for four age groups.

Figure 3 shows the GALM analysis using B-spline. The GALM model is more useful for predicting the probability of occurrence than the generalized linear model. The findings indicate that the rate of occurrence in patients in different age groups differs significantly depending on temperature. From the analysis of Figure 3 it is evident that people under the age of 15 are more than twice as likely to contract norovirus compared to those over the age of 15. Especially, the two age groups containing patients under the age of 15 show the highest probability of contracting norovirus when the temperature is near 0 degrees, while the other two age groups, 16–59 years and over 60 years, have the highest probability of being infected by a norovirus when the temperature is between −10 and 0. In other words, the temperature interval in which the highest probability of norovirus occurs was found to be lower for those over 15 years of age compared to those under 15 years of age.

Table 3 lists the maximum incidence rates, maximum rate temperatures, risk intervals, and high-risk intervals of the four age groups. Table 3 shows the temperatures at which patients in each group are vulnerable to norovirus; this information could prove very helpful in preventing norovirus. As noted in the previous section, the proportion of all diarrhea patients aged 0–5 who contract norovirus is 13%, which is significantly higher than the corresponding proportions in all other age groups. The predicted norovirus infection rate among patients aged 0–5 years reaches a maximum of 23.8% at −2.0 °C, which is significantly greater than the overall observed rate of 13.00%. A risk interval above 19.0% corresponds to an average daily temperature between −10.4 °C and 6.4 °C. In addition, a high-risk interval over 21.4% corresponds to an average daily temperature between −7.8 °C and 3.8 °C. The predicted norovirus infection rate among all diarrhea patients aged 6–15 years reaches a maximum of 21.2% at −0.5 °C; this is significantly greater than the overall observed rate of 8.74% for the same group. A risk interval above 17.0% corresponds to an average daily temperature of between −5.7 °C and 4.6 °C. A high-risk interval exceeding 19.1% corresponds to an average daily temperature between −4.1 °C and 3.0 °C. The predicted norovirus infection rate among all diarrhea patients aged 16–59 reaches a maximum of 11.9% at −5.8 °C, which is more than double the overall observed rate of 4.79% for the same group. A risk interval exceeding 9.5% corresponds to an average daily temperature between −14.3 °C and 2.7 °C. The high-risk interval exceeding 10.7% corresponds to an average daily temperature between −11.7 °C and 0.1°C. The predicted norovirus infection rate among all diarrhea patients aged 60 years or older reaches a maximum of 7.7% at −4.6 °C, which is significantly greater than the overall observed rate of 3.37% for the same group. The risk interval exceeding 6.2% corresponds to an average daily temperature between −12.9 °C and 3.6 °C. A high-risk interval, which exceeds 7.0%, corresponds to an average daily temperature between −10.3 °C and 1.1 °C. In particular, the incidence rates in patients aged 0–5 and 6–15 are much greater than the average rates. In this paper, we constructed scenarios with rates greater than the average (13% for 0–5 years old and 8.74% for 6–15 years old) where norovirus occurs as a priority for each age group.

**Figure 4:**
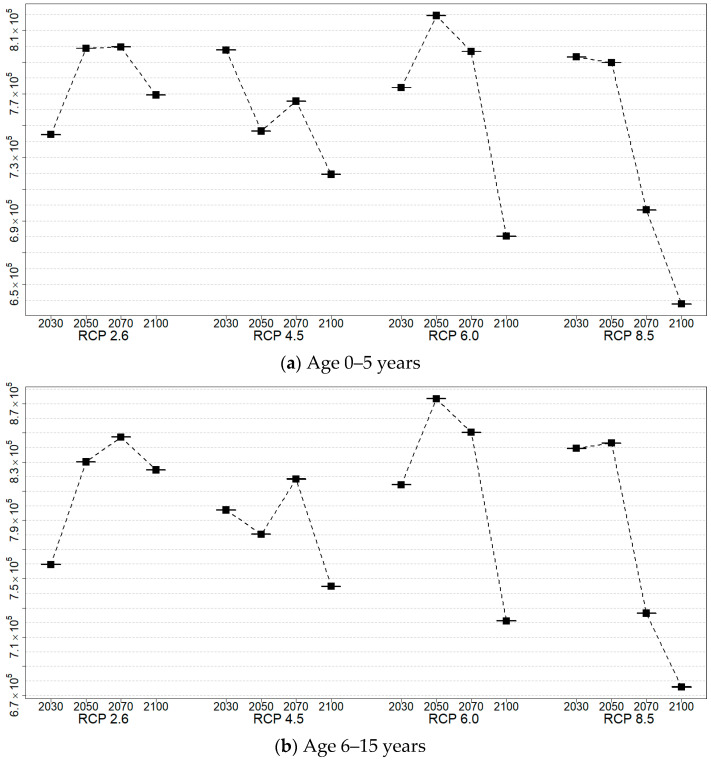

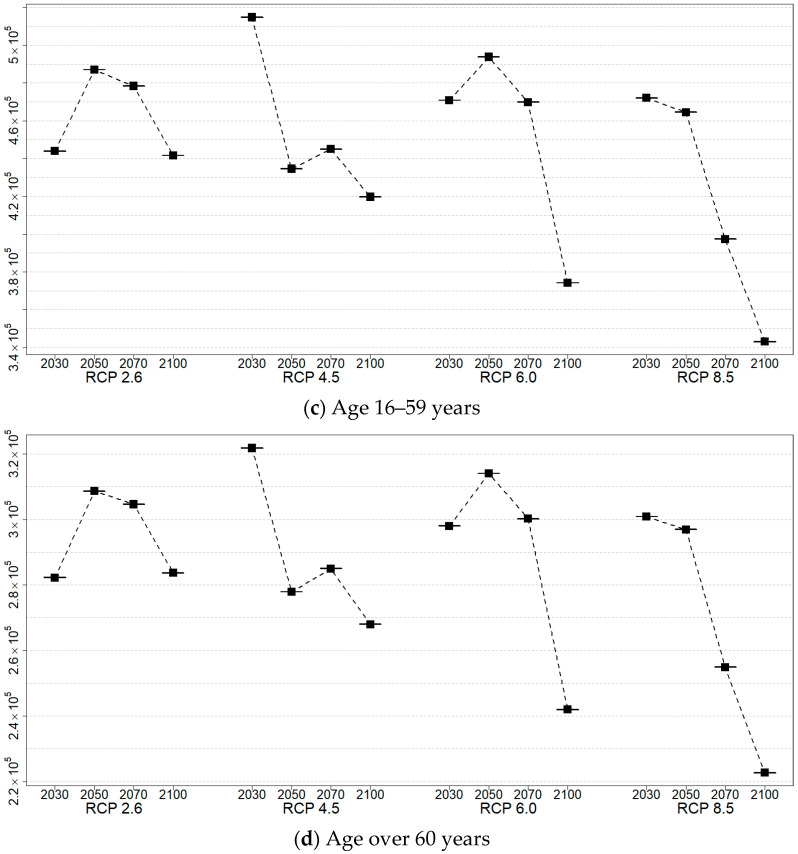
Plots of RRI in 2030, 2050, 2070, and 2100 under four RCP scenarios (RCP2.6, RCP4.5, RCP6.0, and RCP8.5).

**Figure 5:**
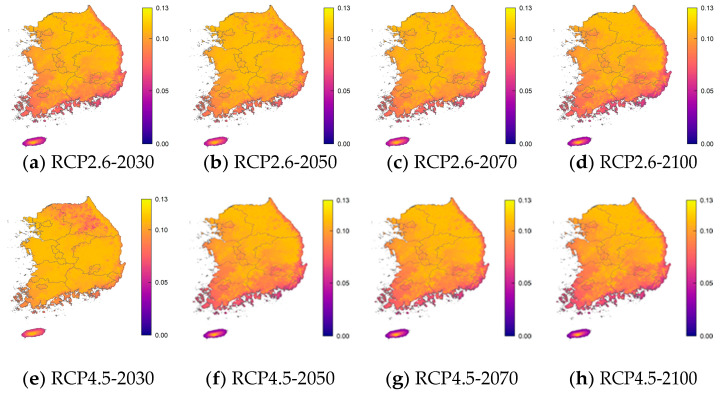

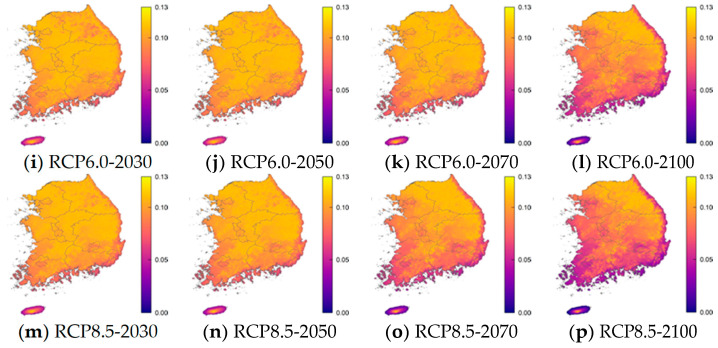
The prediction probability plots of norovirus incidence by the GALM model for Age 0–5 years.

**Figure 6:**
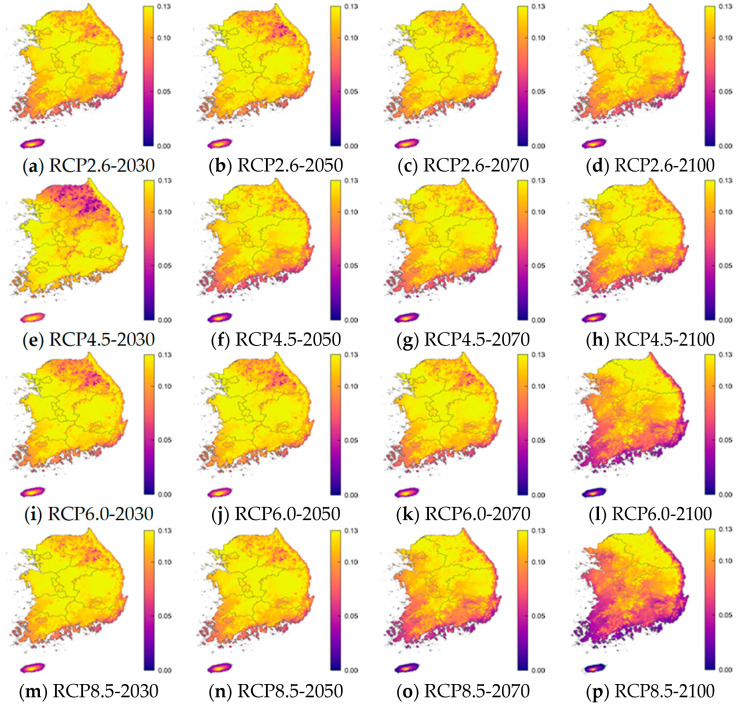
The prediction probability plots of norovirus incidence by the GALM model for Age 6–15 years.

**Figure 7:**
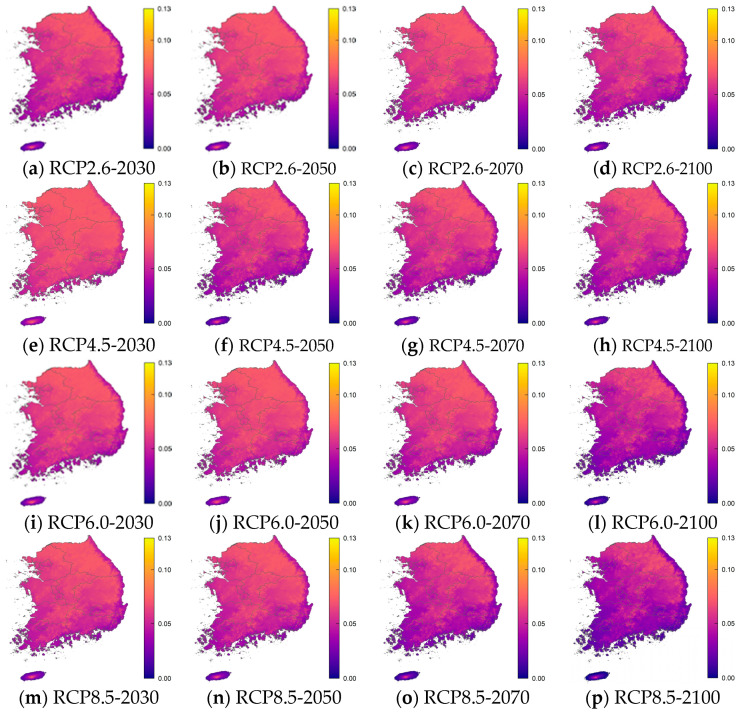
The prediction probability plots of norovirus incidence by the GALM model for Age 16–59 years.

**Figure 8:**
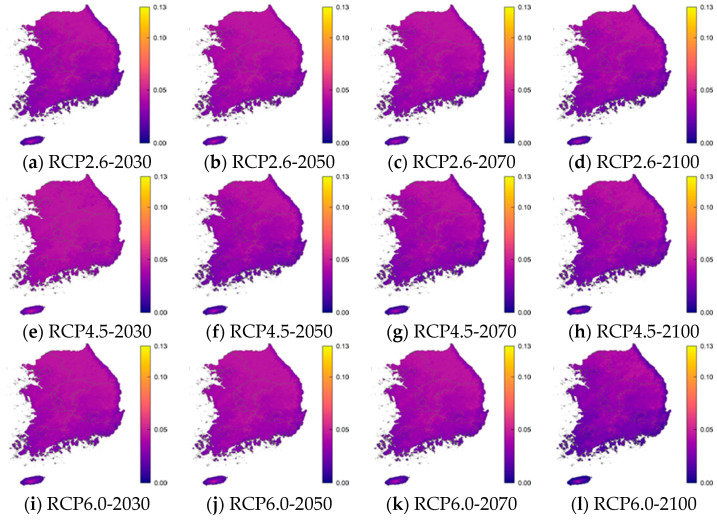

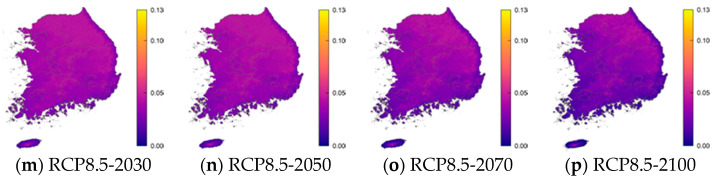
The prediction probability plots of norovirus incidence by the GALM model for Age over 60 years.

For each age group, the RRI was calculated using the critical value of the incidence of norovirus among diarrhea patients. Table 4, Table 5, Table 6 and Table 7 and Figure 4 show the resulting values and 25 and 97.5 percentile of these RRIs by age relative to the critical values in the years 2030, 2050, 2070, and 2100 according to RCP scenarios 2.6, 4.5, 6.0, and 8.5. These results were obtained using percentile bootstrap methods over 100 trials. In Figure 4, the RRI shows a similar pattern in the same age groups and RCP scenarios. In the RCP 2.6 scenario, the RRI for the 0–5 years, 16–59 years, and over 60 years age groups will increase by 2050 and 2070, but in 2100 it is similar or only slightly higher than in 2030. In RCP 4.5, RRI tends to decrease, but it rises briefly in 2070. In RCP 6.0, the RRI increases until 2070 and then decreases slowly, and by 2100 the RRI is decreasing rapidly. In RCP 8.5, the RRI is maintained until 2050, then decreases significantly until 2100. In Figure 5, Figure 6, Figure 7 and Figure 8, we present the probability of being infected with norovirus according to the temperature calculated by GALM for each grid on the map of the Korean Peninsula. Overall, it can be seen that the more rapid the climate change, the lower the probability of infection. In addition, in terms of the regional analysis, the probability of infection decreases from the southern and eastern coasts. In Figure 6, it can be seen that in all RCPs, the probability of infection in the northeast area is low in 2030 and in 2050. It is lower in the coastal areas than in other areas for two age groups, 16 to 60 and over 60 years groups.

## 6. Discussion and Conclusions

In the previous section, we calculated the probability of norovirus occurrence for different age groups by applying the GALM model using average daily temperature as an explanatory variable, and we calculated the risk index according to RCP scenarios based on the RRI index presented in this paper. These results are consistent with the general observation that food poisoning caused by norovirus occurs most often in winter. Considering that the average winter temperature in Korea is 1.5 °C in December, −1.0 °C in January, and 1.1 °C in February, Korea’s winter season is favorable for norovirus to occur. The results show that the risk of norovirus occurrence increases in 2050 and 2070 but decreases by the year 2100 under the RCP 2.6 scenario for 0–5 years, 15–60 years, and over 60 years age groups. For all age groups, the risk of norovirus occurrence decreases over time under the RCP 4.5 scenario, despite a brief increase in 2070. Under the RCP 6.0 scenario, for all age groups, the risk increases until 2070 and then decreases to 2100. Under the RCP 8.5 scenario, for all age groups, the risk remains stable until 2050 and then decreases to 2100. In the prediction probability plots, we can see that the probability of occurrence decreases from near the coast, and that the probability of occurrence in the northeast area is low in 2030 and 2050 in all RCP scenarios. In general, the incidence of norovirus is predicted to decrease when the temperature rises due to climate change. However, the findings of this study indicate that, even if the temperature rises, the temperature in Korea will remain within the risk interval of temperature for norovirus occurrence, so the risk of norovirus will either remain stable or increase. Despite the predicted increase in temperature due to climate change, it is necessary to continue to prepare for future norovirus outbreaks in Korea, among those under the age of 15. Based on these simulation results, if the winter temperature rises, the possibility of food poisoning due to domestic norovirus will increase, and effective disease control measures will be needed. Of course, our ability to predict changes in the pattern of norovirus outbreaks simply by looking at the average daily temperature alone may be somewhat limited. A number of factors should be considered to improve the accuracy of these predictions, such as humidity and precipitation in particular, as these are closely related to microbial reproduction. In fact, there have been reports that lower water temperatures increase the viability of noroviruses in their natural environments [39,40], and, statistically, lower water temperatures in the Ontario River have been associated with many cases of food poisoning caused by noroviruses [41]. Norovirus can be transmitted not only through fecal and oral routes but also through aerosols caused by vomiting, so an increase in humidity is likely to increase pathogen viability [40]. In a similar vein, there have been reports that high humidity increases the viability of rotavirus [42]. In order to reasonably predict changes in the seasonal patterns of norovirus outbreaks, it is necessary to consider not only climate-related factors but also human behavior and socioeconomic changes that may occur with climate change. The cooler temperatures in winter cause people to remain inside longer, thus increasing the incidence of food poisoning from norovirus, meaning that changes in human behavior can affect the incidence of norovirus.

This paper calculated the risk index according to the temperature changes suggested in various RCP scenarios, but this approach has limitations, as the occurrence of norovirus is affected by other latent factors. For example, outbreaks of food poisoning caused by norovirus are often related to the consumption of fresh fruits and vegetables, so a significant increase in the consumption of fresh fruits and vegetables in winter could lead to a marked increase in norovirus food poisoning regardless of the climate conditions. In addition, 42.5% of cases of food poisoning caused by norovirus in Korea from 2000 to 2007 are known to have been caused by careless food handling by cooks, so improved hygiene practices among individuals working in large centers that handle fresh agricultural products could reduce incidences of norovirus. Nevertheless, based on the results of this study, we predict that the risk of norovirus in winter will either remain stable or increase in Korea despite climate change leading to warmer winters. This approach provides an overview of the likely changes that will occur in regional and seasonal norovirus patterns, which are expected to help appropriate policy decisions.

## Figures and Tables

**Table 1:** Clinical characteristics of Korean patients with diarrhea (2005–2018).

Characteristic		Number of Patients with Norovirus(%)	Total Diarrhea Patients
Total		24,642 (8.69)	283,651
Gender	Male	13,787 (8.82)	156,397
	Female	10,855 (8.53)	127,254
Age	0–5	17,390 (13.00)	133,737
	6–15	2062 (8.74)	23,605
	16–59	3139 (4.79)	65,524
	over 60	2051 (3.37)	60,785
Region	South Area	11,706 (9.26)	126,467
	Central Area	4452 (6.05)	73,533
	North Area	8484 (10.14)	83,651

**Table 2:** Results of logistic regression analysis.

Covariates		*β*	exp (*β*)	se	*p*-Value	95% CI
Intercept		−1.884	0.152	0.012	<0.001	0.148–0.156
Gender	Male			Reference		
	Female	0.01	1.01	0.014	0.461	0.983–1.037
Age	0–5			Reference		
	6–15	−0.437	0.646	0.024	<0.001	0.616–0.678
	16–59	−1.051	0.350	0.02	<0.001	0.336–0.364
	over 60	−1.405	0.245	0.024	<0.001	0.234–0.257
Region	South Area			Reference		
	Central Area	−0.252	0.777	0.019	<0.001	0.749–0.806
	North Area	0.06	1.062	0.015	<0.001	1.031–1.094

**Table 3:** Calculated maximum norovirus incidence rate and temperature by age group.

Age	Maximum Rate	Maximum Rate Temperature	Risk Interval			High-Risk Interval		
			Rate	Temperature Range		Rate	Temperature Range	
0–5	23.8%	−2 °C	19.0%	−10.4 °C	6.4 °C	21.4%	−7.8 °C	3.8 °C
6–15	21.2%	−0.5 °C	17.0%	−5.7 °C	4.6 °C	19.1%	−4.1 °C	3 °C
16–59	11.9%	−5.8 °C	9.5%	−14.3 °C	2.7 °C	10.7%	−11.7 °C	0.1 °C
Over 60	7.7%	−4.6 °C	6.2%	−12.9 °C	3.6 °C	7.0%	−10.3 °C	1.1 °C

**Table 4:** RRI for the 0–5 years age group in the four RCP scenarios.

Age	RCP	Year	RRI	se	2.5%	97.50%
0–5	RCP2.6	2030	744,363.191	56.249	744,238.988	744,475.598
		2050	798,557.165	58.093	798,448.003	798,682.025
		2070	799,461.843	58.131	799,364.242	799,581.800
		2100	769,219.313	64.379	769,104.059	769,342.982
	RCP4.5	2030	797,518.059	50.979	797,417.997	797,620.212
		2050	746,507.322	54.491	746,404.296	746,603.098
		2070	765,325.678	66.426	765,193.106	765,443.671
		2100	719,243.670	72.033	719,098.98	719,370.906
	RCP6.0	2030	773,885.898	59.472	773,778.616	774,007.359
		2050	819,199.541	45.961	819,120.154	819,287.547
		2070	796,606.392	48.595	796,516.908	796,685.359
		2100	680,299.531	78.89	680,118.010	680,425.951
	RCP8.5	2030	793,144.660	52.257	793,056.644	793,233.400
		2050	789,608.884	58.676	789,479.547	789,718.438
		2070	696,860.635	77.764	696,731.282	697,019.472
		2100	637,696.714	76.576	637,551.772	637,827.478

**Table 5:** RRI for the 6–15 years age group in the four RCP scenarios.

Age	RCP	Year	RRI	se	2.5%	97.50%
6–15	RCP2.6	2030	759,573.415	87.567	759,392.791	759,752.307
		2050	830,048.756	87.292	829,897.173	830,171.827
		2070	847,114.171	85.711	846,989.130	847,286.762
		2100	824,541.476	85.232	824,408.661	824,737.405
	RCP4.5	2030	797,031.139	92.947	796,868.583	797,198.477
		2050	780,405.365	88.667	780,244.899	780,579.088
		2070	818,259.560	91.263	818,110.020	818,439.662
		2100	744,777.347	109.334	744,567.330	744,988.180
	RCP6.0	2030	814,398.394	76.988	814,255.317	814,547.288
		2050	873,433.728	78.062	873,283.645	873,577.149
		2070	850,347.595	85.542	850,199.707	850,538.008
		2100	721,090.103	102.792	720,891.629	721,264.156
	RCP8.5	2030	839,317.621	80.411	839,185.498	839,501.208
		2050	842,927.700	82.334	842,793.436	843,091.020
		2070	726,317.494	119.128	726,139.886	726,580.614
		2100	675,713.129	112.328	675,513.487	675,906.920

**Table 6:** RRI for the 16–59 years age group in the four RCP scenarios.

Age	RCP	Year	RRI	se	2.5%	97.50%
16–59	RCP2.6	2030	443,918.399	56.697	443,813.478	444,020.317
		2050	487,006.047	42.343	486,927.932	487,083.852
		2070	478,341.416	36.410	478,277.169	478,404.044
		2100	441,578.840	46.270	441,503.240	441,667.933
	RCP4.5	2030	514,711.088	38.449	514,641.502	514,797.870
		2050	434,542.392	49.690	434,429.309	434,637.481
		2070	444,944.118	50.705	444,840.469	445,052.900
		2100	419,674.710	55.697	419,577.711	419,794.594
	RCP6.0	2030	470,768.449	46.386	470,685.615	470,851.935
		2050	493,738.981	38.454	493,661.984	493,798.576
		2070	469,803.798	44.892	469,712.005	469,881.028
		2100	374,173.545	60.717	374,068.956	374,298.359
	RCP8.5	2030	472,046.924	47.130	471,954.943	472,142.872
		2050	464,492.952	47.240	464,407.449	464,577.836
		2070	397,364.829	63.693	397,242.417	397,481.481
		2100	343,059.165	56.321	342,955.761	343,160.810

**Table 7:** RRI for those aged more than 60 years in the four RCP scenarios.

Age	RCP	Year	RRI	se	2.5%	97.50%
over 60	RCP2.6	2030	282,203.504	26.677	282,156.802	282,257.290
		2050	308,661.955	26.281	308,604.289	308,703.138
		2070	304,635.017	25.072	304,589.212	304,683.773
		2100	283,677.657	28.946	283,622.792	283,730.317
	RCP4.5	2030	321,812.213	20.200	321,776.156	321,848.276
		2050	277,891.568	30.253	277,824.773	277,943.525
		2070	284,957.856	33.514	284,888.233	285,012.275
		2100	267,956.163	31.890	267,900.299	268,015.764
	RCP6.0	2030	298,005.491	26.261	297,947.565	298,047.385
		2050	314,090.960	21.365	314,052.541	314,135.816
		2070	300,210.740	29.942	300,160.387	300,264.328
		2100	241,975.568	36.335	241,903.235	242,032.298
	RCP8.5	2030	300,852.815	24.281	300,802.806	300,888.892
		2050	296,927.581	26.964	296,875.699	296,971.395
		2070	254,937.229	35.084	254,858.145	254,992.024
		2100	222,659.981	35.031	222,595.774	222,712.245
